# Shared Cultural Values Influence Mental Health Help-Seeking Behaviors in Asian and Latinx College Students

**DOI:** 10.1007/s40615-021-01073-w

**Published:** 2021-06-23

**Authors:** E. Zhou, Y. Kyeong, C. S. Cheung, K. J. Michalska

**Affiliations:** Department of Psychology, University of California Riverside, 900 University Ave. Riverside, California, CA 92521 USA

**Keywords:** Culture, Values, Help-seeking, Mental health attitudes, College students

## Abstract

The current study sought to understand the influence of cultural values on mental health attitudes and help-seeking behaviors in college students of diverse ethnic backgrounds. Asian and Latinx college students (*N* = 159) completed an online survey in which they reported on their adherence to cultural values as measured by ethnicity-specific cultural values and general attitudes towards mental health. Factor analysis revealed two common factors of cultural values irrespective of ethnicity: *Interdependent Orientation* (IO) and *Cultural Obligation* (CO). Regardless of ethnicity, the more students endorsed IO values, the less likely they were to perceive a need for mental health treatment. IO value adherence was also predictive of more negative attitudes towards mental health. CO values were not predictive of perceived need or help-seeking behaviors. Findings highlight the importance of understanding shared cultural values across ethnic-racial groups and considering how the multidimensionality of culture may help explain shared mental health behaviors crossing lines of ethnic group membership.

**Supplementary Information:**

The online version contains supplementary material available at 10.1007/s40615-021-01073-w.

National rates of student mental health diagnoses and service utilization are increasing on college campuses across the USA [1], yet glaring racial-ethnic disparities exist in treatment-seeking behavior and receipt of psychological services [2]. Despite higher rates of psychological distress reported by Asian and Latinx students in particular [2, 3], these students access campus mental health care services at lower rates than their non-Latinx White peers [4, 5]. Of course, ethnic group membership alone does not determine whether a student will seek mental health services. Other underlying factors need to be examined to understand how members of these groups approach available campus resources. We focus here on the role of cultural values pertinent to these ethnic groups on students’ mental health help-seeking behaviors, complementing the extensive work on external barriers to seeking treatment including language or financial resources [6–9]. Though existing work has drawn connections between cultural values and mental health attitudes among both Asian and Latinx groups, the present work furthers the literature by examining shared values across groups [10, 11]. Specifically, this study examined whether family- and group-oriented dimensions of cultural values that are shared among Asian and Latinx college students are associated with perceived need for mental health treatment and mental health help-seeking, above demographic characteristics. A focus on shared cultural values can help mental health service providers understand barriers to help-seeking more completely without assigning blame to any specific ethnicity or culture.

## Familism and Mental Health Behaviors

Individuals from Latinx [12] and Asian [13] cultures value *familism*, or the duty to assist, support, and respect one’s family. Research conducted separately in both groups has established associations between high levels of familism and improved well-being via supportive family ties [14, 15]. However, because family is viewed as an extension of the self in both cultures and the needs of the family are valued above the needs of the individual [16, 17], this can also imply that if an individual is experiencing psychological distress, then shame or stigma faced by that individual may also be experienced by the family. In line with this notion, acknowledging mental health problems is sometimes perceived as a failure shared by the family and extended kinship group in these cultures [18, 19]. Familism therefore may foreshadow unwillingness to seek professional treatment for mental illness [20, 21]. Familism in Latinx households has also been associated with increased informal and religious avenues of help-seeking for mental health care but not specialty mental health service use [22]. Ultimately, even though familism can be protective for mental health of youth from Asian and Latinx backgrounds, it may diminish some forms of mental health help-seeking behaviors in these youth. Of note, previous work has observed gender differences in the associations between familism and mental health outcomes among individuals of Asian and Latinx backgrounds, with women reporting poorer psychological health than men, despite greater levels of familism and support [14]. A study by Sanchez and Atkinson similarly finds that though strong Mexican-American cultural commitment was related to lesser willingness towards self-disclosure in counseling overall, females were more willing to use counseling services than males [23]. In another study with Asian college students, Gim, Atkinson, and colleagues also observed a relationship between willingness to seek counseling and both gender and acculturation [24]. However, though the gender difference was consistent such that women were more willing to seek counseling than men, the relationship with acculturation was not, where by those individuals who reported lower levels of acculturation were more open to professional help-seeking than those who reported higher levels of acculturation. The finding that less acculturated Asian students were more willing to see a counselor directly contradicted a previous study from Atkinson and Gim, which found that in a sample of Asian students, those most acculturated held the most positive attitudes towards mental health services [25]. Given these mixed findings, it is important to consider how cultural values and gender might independently and interactively predict mental health behaviors across different groups in the college environment. To our knowledge, no study has examined this question among Asian and Latinx college students.

## Mental Health Attitudes as Barriers to Mental Health Help-Seeking

### Mental Health Stigma

One prominent psychological barrier to mental health help-seeking is mental health stigma, which manifests primarily in social and self-stigma, or negative public attitudes towards mental illness and the internalization of these public perceptions [26, 27]. Asian and Latinx students generally report greater stigma about mental illness and mental health help-seeking behaviors compared with non-Latinx White students [28, 29], which may in part be due to mental illness being considered an admission of emotional weakness [30, 31]. Students of both Asian and Latinx heritage also differ from those of European heritage in their appraisals of negative emotions, deeming them more undesirable and inappropriate to experience and express [32]. Stigma and shame, in conjunction with viewing the family as an extension of the self, may lead to resistance towards mental health treatment-seeking, as acknowledging a need for mental health treatment may reflect poorly on the individual and their family. Both Asian and Latinx groups value the avoidance of shame [33, 34], and seeking treatment for mental health issues may be perceived as particularly shameful. To avoid bringing shame to themselves and their families, Asian and Latinx college students may therefore shy away from initiating mental health treatment [35, 36]. One particularly alarming example of the adverse effects of stigma was documented among a sample of suicidal Latina adolescents, who viewed suicide as a viable solution to alleviate undue burdens of shame on their families [37].

Studies that have directly evaluated links between sociocultural values and mental health help-seeking stigma have yielded mixed findings. Even though several such studies find positive associations between Asian cultural values and stigma, not all indicate that stigma is directly relevant to help-seeking behavior [38, 39]. For example, Kim & Zane (2016) found that even though Asian American students perceived greater barriers to help-seeking than non-Latinx White students, both groups showed an inverse relationship between stigma and help-seeking intentions [40]. Likewise, in the literature on Latinx cultural values and stigma, adults report that stigma resulting from cultural expectations discourages them from admitting need for mental health help and adhering to a treatment plan, though less is known about its relationship to seeking psychological services in college students [30]. Some researchers find a stronger relationship with Latinx cultural values and stigma in older adults and a weaker one in college students, but it is yet unclear how this relationship might influence mental health help-seeking [41, 42]. Collectively, these findings highlight both the complex role of cultural values in mental health contexts and the need for additional research in culturally diverse settings.

### Perceptions of Mental Illness

Beyond stigma, diminished rates of help-seeking in both Asian and Latinx college students may also stem from differing perceptions of what constitutes a mental illness warranting professional treatment. Both ethnic groups experience somatization, reporting physical ailments in lieu of mental illness [43, 44]. The tendency towards somatization may inadvertently stem from stigma, in that it is more culturally acceptable to experience and seek treatment for physical ailments over mental ones [45]. In fact, when somatic symptoms emerge, Asian and Latinx individuals often seek out help from medical doctors or alternative healers instead of psychological professionals for treatment [46–49], potentially leading to greater rates of misdiagnosis [50]. Additionally, those Asian and Latinx individuals who seek professional psychological treatment often present with more severe symptoms and tend to experience problems for a prolonged period of time prior to treatment, suggesting that the threshold for seeking mental health services is higher for them [51–53]. Based on the mental health attitudes largely shared by Asian and Latinx college students, they may be similarly deterred from making initial contact with on-campus mental health service providers. Yet, scant research has examined the shared cultural values that may influence mental health utilization behaviors across ethnic-racial groups.

## The Present Study

Mental health treatment-seeking behaviors of Asian and Latinx students have largely been examined within-group, even though different studies find that similar cultural values influence help-seeking behaviors in both Asian and Latinx youth [6, 35]. Despite significant heterogeneity across and within cultures, we contend that some shared cultural values may lead to less utilization of campus mental health services by Asian and Latinx students. Importantly, we do not put forth that Western mental health services are the only intervention of choice for all students. However, given that available treatments in university settings in the USA are traditional Western psychological treatment services, it is important to more comprehensively understand students’ engagement with such programs. The present study therefore aimed to examine shared relationships between cultural values, mental health attitudes, and mental health behaviors (perceived mental health treatment need and help-seeking) in Asian and Latinx college students. Specifically, we tested (a) the extent to which cultural values endorsed by Asian and Latinx college students were shared, and how cultural values predicted (b) attitudes toward mental health, and (c) perception of mental health treatment need and help-seeking behaviors, above ethnicity alone.

## Method

### Participants

One hundred fifty-nine undergraduate college students (61% female, 58.5% Asian, 41.5% Latinx, 55.3% first-year students) from a public university in Southern California completed a six-section online survey hosted on Qualtrics. The sample was representative of the demographic majority of the university and California broadly [54], with about 75.3% of students and 54% of California residents identifying as Asian or Latinx in 2019, respectively. All participants were recruited from the psychology department participant pool. Consent was obtained from all participants via electronic acknowledgment. Students were awarded course credit for participation. Study approval was obtained from the university Institutional Review Board.

### Measures

#### Cultural Values

##### Asian Values Scale - Revised (AVS-R)

The *Asian Values Scale-Revised* (AVS-R) [55] is a 25-item measure assessing endorsement of values associated with the Asian culture shortened from the original 36-item measure [56]. Participants indicated the extent to which each statement applied to them (1 = strongly disagree; 4 = strongly agree), with higher scores indicating greater adherence to Asian cultural values. Example items include “One should consider the needs of others before considering one’s own needs” and “One should not deviate from familial and social norms.” The scale has demonstrated strong reliability and validity in past research in university student samples [55], and Cronbach’s alpha in this sample was .81.

##### Latino Values Scale (LVS)

Participants also completed the 35-item *Latino Values Scale* (LVS) [57] measuring endorsement of values associated with the Latinx culture. Participants indicated the extent to which each statement applied to them (1 = strongly disagree; 4 = strongly agree), with higher scores indicating greater adherence to Latinx cultural values. Example items include “One’s family is the main source of one’s identity” and “One must not offend others.” The measure yields a total score (LVS - Total) and two subscale scores (LVS – “Cultural Pride” and LVS – “Familismo”). The scale has demonstrated strong reliability and validity in past research in university student populations [57]. We assessed adherence to Latinx values via the LVS - Total, for which the Cronbach’s alpha in this sample was .89.

#### Attitudes Towards Mental Health

##### Attitudes Towards Mental Illness (AMI)

Mental health attitudes were assessed using 47 items adopted from two scales: *Community Attitudes Toward Mental Illness* (CAMI) [58] and *Reported and Intended Behaviour Scale* (RIBS) [59].

The CAMI is a 40-item measure that assesses prejudice towards and exclusion of the mentally ill and tolerance/support for community care. Example items include, “One of the main causes of mental illness is a lack of self-discipline and willpower” and “Mental illness is an illness like any other.” Following prior recommendations (UK Department of Health Attitudes to Mental Illness Questionnaire) [60], we retained 26 of the original 40 items and added one item on employment attitudes. To facilitate interpretation, negative items were reverse-coded, such that overall scores indicated positive attitudes towards mental illness. Cronbach’s alpha for the CAMI in this sample was .86.

The RIBS is an 8-item instrument that measures both reported engagement (e.g., “Are you currently living with, or have you ever lived with, someone with a mental health problem?”) and intended engagement (e.g., “In the future, I would be willing to live with someone with a mental health problem”) with individuals with mental health problems. To measure students’ mental health attitudes, four items specifically assessing intended engagement were used. Cronbach’s alpha for the RIBS in this sample was .88.

Both the CAMI and the RIBS scales were scored based on agreement with correct or non-stigmatizing statements on a 5-point Likert scale (1 = strongly disagree; 5 = strongly agree). Because of substantial correlation between the two scales (*r* = 0.65, *p* < 0.001), a composite score was used. Low scores on the AMI composite score correspond to negative attitudes, and high scores correspond to positive attitudes. Cronbach’s alpha for the composite score in our sample was .89.

##### Perceived Mental Health Service Need and Help-Seeking Behavior

Participants also responded to two multiple-choice questions about their own mental health needs and help-seeking behavior. First, they responded either “Yes” or “No” to the question, “Do you feel you need mental health help?” Only those who responded affirmatively (*n* = 33) were then asked to respond “Yes” or “No” to “Are you seeking treatment?”

### Data Analytic Plan

The aims of this research were to determine whether some cultural values are shared among Asian and Latinx college students and whether the degree of adherence to these values predicts students’ attitudes to mental health and their perceived treatment need. To address the first aim, we conducted an exploratory factor analysis of all items across Asian and Latino value scales (AVS-R and LVS respectively, see Table S1 for details), without an a priori hypothesis regarding the number of resultant factors. To address the second aim, we performed a series of linear regression analyses to test whether AVS-R and LVS predicted students’ attitudes to mental health. We then repeated these analyses, using the factors that emerged from our factor analysis as predictors of attitudes to mental health. To further shed light on the association between shared cultural values and students’ help-seeking behaviors, we conducted hierarchical logistic regressions to test whether shared cultural values predicted students’ perceived treatment need and help-seeking behaviors. Given our focus on cultural values above and beyond ethnic group membership, all regressions controlled for ethnicity. Participant gender was also included as a covariate to account for potential gendered cultural expectations, particularly pertaining to familism. In logistic regression models examining mental health behaviors, mental health attitudes were covaried to isolate the unique effects of cultural values on mental health behaviors.

## Results

Descriptive statistics and zero-order correlations among all study variables are presented in Table 1. Overall, even though Asian and Latinx values were correlated, both Asian and Latinx students endorsed values in the Latinx values scale (*M* = 2.50, *SD* = 0.32) to a greater extent than those in the Asian values scale (*M* = 2.35, *SD* = 0.31), *t*(158) = −8.61, *p* < 0.001. An independent-samples *t*-test revealed that Asian students endorsed Asian values significantly more (*M* = 2.43, *SD* = 0.27) than did Latinx students (*M* = 2.24, *SD* = 0.33), *t*(157) = 3.91, *p* < 0.001; there was no significant difference between groups in endorsement of Latinx values.

**Table 1:** Descriptive statistics and zero-order correlations for key study variables

Variable	*M*	*SD*	1	2	3
1. LVS	2.50	0.32	**-**	0.75***	−0.29***
2. AVS-R	2.35	0.31		-	−0.49***
3. AMI	118.37	14.62			-

Scaled LVS and scaled AVS-R scores have a possible range from 1 to 4; AMI scores from 30 to 150

*LVS* Latino Values Scale, *AVS-R* Asian Values Scale-Revised, *AMI* Attitudes to Mental Illness

***p < 0.001

### Analysis One: Common Factor Structure among Asian and Latinx Value Scale Items

We first conducted an exploratory factor analysis, combining all items within both the AVS-R and the LVS into one model to examine if latent variables were driving students’ response patterns to both scales. To this end, we tested whether items loaded onto similar factors across the two groups from the pool of the 25 AVS-R items and 35 LVS items (Table S1).

Our analysis revealed two underlying factors, which we labeled *Interdependent Orientation* (IO, 7 items) and *Cultural Obligation* (CO, 7 items). Items in the IO factor centered around the importance of one’s family and in-group, whereas items in the CO factor focused on the importance of actively participating in and preserving one’s culture. Each of the factors was internally consistent, with a Cronbach’s alpha of .77 for IO and .83 for CO. The two factors were positively correlated (*r* = 0.35, *p* < 0.001) within the whole group and within each ethnic group (Asian: *r* = 0.39, *p* < 0.001, Latinx: *r* = 0.31, *p* = 0.01). Independent-samples *t*-tests revealed no significant difference in mean scores of IO between participant groups (*p* = 0.73) and significantly greater mean scores on CO for Asian participants (*M* = 2.73, *SD* = 0.46) than Latinx (*M* = 2.58, *SD* = 0.51) participants, *t*(157) = 2.01, *p* < 0.05.

### Analysis Two: Associations Between Value Endorsement and Attitudes to Mental Health and Help-Seeking

Next, we used three sets of hierarchical linear regressions to examine the AVS-R, LVS, and the two shared factors derived in the previous set of analyses, as predictors of students’ attitudes to mental health. A first set of hierarchical linear regressions examined the AVS-R as a predictor of attitudes towards mental health. A second set of hierarchical linear regressions examined the LVS as a predictor of attitudes towards mental health. These analyses revealed that both the AVS-R and the LVS added significantly to the prediction of students’ attitudes to mental health (see supplementary materials).

A third set of hierarchical linear regressions examined the two shared factors that emerged from our factor analysis (i.e., IO, CO) as predictors of students’ attitudes towards mental health. Using attitudes to mental health as the dependent variable in two separate stepwise regressions, we entered ethnicity and gender in an initial step, either the IO factor or the CO factor in a second step, and factor × demographic interaction terms in a third step.

In the analysis focusing on the IO factor, ethnicity and gender were significantly associated with attitudes towards mental health, *R*^2^ = 0.12, *F*(2,156) = 10.91, *p* < 0.001. The IO factor significantly added to the prediction of attitudes towards mental health, Δ*R*^2^ = 0.07, *F*(1,155) = 13.02 , *p* < 0.001. IO was a significant inverse predictor of the AMI, after controlling for demographic variables. The step containing the interaction term with the demographic variables did not add significantly to the prediction of AMI (see Table S4).

In the analysis focusing on the CO factor, ethnicity and gender were significantly associated with attitudes towards mental health, *R*^2^ = 0.12, *F*(2,156) = 10.91, *p* < 0.001. The CO factor did not add significantly to the prediction of attitudes towards mental health. Similarly, the interaction term with ethnicity and gender did not add significantly to the prediction of attitudes towards mental health (see Table S5).

### Analysis Three: Associations Between Cultural Value Endorsement and Perceived Treatment Need and Help-Seeking

Three sets of hierarchical logistic regressions were conducted to examine whether students’ attitudes towards mental health and their endorsement of cultural values predicted their perceived treatment need and their help-seeking behaviors. First, we tested whether students’ mental health attitudes (assessed via the AMI) and their endorsement of Asian values (assessed via the AVS-R) predicted the likelihood that they perceived a need for treatment or the likelihood of their mental health help-seeking behaviors. Results are shown in Table S6. After controlling for gender and ethnicity, AMI (OR = 1.05, 95% CI = [1.01, 1.08]) and AVS-R (OR = 0.08, 95% CI: [0.01, 0.98]) predicted the likelihood that students perceived a need for mental health treatment. Specifically, for each unit increase in positive attitudes toward mental health, students were 5% more likely to perceive needing mental health treatment (*β* = 0.04, *p* = 0.01). In contrast, for each unit increase in adherence to Asian values, students were 92% less likely to perceive a need for mental health treatment (*β* = −2.59, *p* < 0.05). No significant predictors emerged with help-seeking behavior as the outcome variable (*p* > 0.27).

The second set of logistic regressions examined whether students’ mental health attitudes (assessed via the AMI) and their endorsement of Latinx values (assessed via the LVS) predicted the likelihood of their perceived treatment need and help-seeking behavior. Results are shown in Table S7. After controlling for gender and ethnicity, a main effect of AMI emerged in predicting the likelihood of perceived treatment need (OR = 1.06, 95% CI = [1.02, 1.09]), whereby for each unit increase in positive attitudes towards mental health, students were 6% more likely to perceive a need for treatment (*β* = 0.05, *p* < 0.01). No significant predictors emerged with help-seeking behavior as the outcome variable (*p* > 0.26).

The third set of logistic regressions examined whether our two factors, IO and CO, predicted students’ perceived treatment need and their help-seeking behavior. Ethnicity and gender were entered in step 1, AMI was entered in step 2, IO and CO were entered in step 3, and factor × demographic interactions were entered in step 4. Results showed main effects of AMI (OR = 1.05, 95% CI = [1.02, 1.09]) and our IO factor (OR = 0.10, 95% CI = [0.02, 0.55]) on the likelihood of perceived treatment need. For each unit increase in positive attitudes to mental health, students were 5% more likely to perceive a need for treatment (*β* = 0.05, *p* < 0.01). Conversely, for each unit increase in IO, students were 90% less likely to perceive a need for treatment (*β* = −2.30, *p* < 0.01). With help-seeking behavior as the outcome variable, only the interaction between IO and gender was significant (OR = 0.00, 95% CI = [0.00, 0.74]) (Table 2). Because the main effect of IO was not significant and the sample size reporting on help-seeking was small, we did not probe this interaction further.

Our final analysis looking at the CO factor revealed only a main effect of AMI (OR = 1.07, 95% CI = [1.03, 1.10]) on the likelihood of perceived treatment need. For each unit increase in positive attitudes to mental health, students were 7% more likely to perceive a need for treatment (*β* = 0.06, *p* < 0.001). No significant predictors emerged with help-seeking behavior as the outcome variable (*p* > 0.12) (Table S8).

**Table 2:** Logistic regressions with interdependent orientation predicting perceived need and help-seeking behavior

Outcome		Perceived need								Help-seeking	
	Predictor	OR	*SE*	95% CI		*p*	OR	*SE*	95% CI		*p*
				*LL*	*UL*				*LL*	*UL*	
Step 1	Ethnicity^a^	0.48	0.50	0.18	1.28	0.14	49.05	2.36	0.48	5046.28	0.10
	Gender^b^	0.70	0.54	0.24	2.01	0.51	0.01	2.51	0.00	1.62	0.08
Step 2	AMI	1.05	0.02	1.02	1.09	<0.01	1.03	0.04	0.95	1.12	0.50
Step 3	IO	0.10	0.87	0.02	0.56	<0.01	47.85	2.55	0.32	7094.12	0.13
Step 4	IO × ethnicity	2.94	1.08	0.35	24.48	0.32	376.32	5.60	0.01	21811208.50	0.29
	IO × gender	6.46	1.06	0.80	51.94	0.08	0.00	4.91	0.00	0.74	0.04

*CI* 95% confidence interval, *LL* lower limit, *UL* upper limit, *AMI* attitudes towards mental illness, *IO* interdependent orientation factor, *OR* odds ratio

^a^0 = Hispanic, 1 = Asian

^b^0 = female, 1 = male

## Discussion

Deteriorating mental health among ethnic minority students, along with a reluctance on the part of these students to seek out available behavioral health services, is a growing concern on many college and university campuses. Prior research has examined the role of culture as a barrier to mental health help-seeking among individuals from Asian and Latinx backgrounds. However, this work largely overlooks the possibility that shared cultural values may jointly predict mental health outcomes across ethnic-racial subgroups. Departing from a focus on investigating determinants of help-seeking behaviors within the confines of individual ethnic group boundaries, the current study focused on identifying shared cultural values that might deter *both* Asian and Latinx college students from seeking mental health treatment. Three main findings emerged. First, factor analyses revealed that students from both Asian and Latinx backgrounds endorsed two shared cultural values: *Interdependent Orientation*, a prioritization of respecting and supporting one’s family and in-group, and *Cultural Obligation*, a duty to maintain and participate in one’s broader culture. Second, irrespective of students’ ethnicity, the factor-analytically derived *Interdependent Orientation* value predicted students’ mental health attitudes. Third, *Interdependent Orientation* was an inverse predictor of perceived mental health need, over and above students’ ethnic backgrounds. Our findings point to the intriguing possibility that despite heterogeneity among Asian and Latinx students, shared cultural values are predictive of both mental health attitudes and behaviors and thus warrant special attention in college counseling and other mental health treatment settings.

The current study, to our knowledge, is the first to investigate the association between shared cultural values and mental health help-seeking behaviors in college students across ethnic backgrounds. Interdependent values consistently predict mental illness stigma and avoidance of help-seeking in both Asian and Latinx populations [61], yet open questions remain with regards to how we conceptualize values traditionally considered culturally specific. For instance, is it important that values associated with distinct cultural groups be tied to one particular ethnicity, or can shared cultural values also inform our understanding of mental health behaviors? We thus attempted to unpack elements of group variables that contribute to the relationship between ethnic group status and mental health help-seeking. We observed that values typically believed to be associated with specific ethnicities, when assessed across multiple ethnicities, influenced perceived treatment need above and beyond the effect of ethnicity. Our findings suggest the necessity to reconsider which students should be deemed “at-risk.” Understanding how adherence to certain cultural values may serve as a risk factor for poor help-seeking behavior is important from a practical standpoint. By decoupling ethnic group and culture-specific values, we may be able to strengthen approaches to better serve students in ethnically diverse campuses.

### Cultural Values and Mental Health Attitudes

Even though the IO and CO factors were derived from scales designed specifically to measure Asian and Latinx cultural values, the factors differentially predicted attitudes to mental health and help-seeking. Our finding that greater IO was predictive of more negative attitudes towards mental health across groups is consistent with prior research showing that in-group orientation and familism are associated with mental health stigma [22]. In contrast, greater CO did not predict attitudes to mental health or to help-seeking behavior. Given that the IO factor highlights the in-group and family unit, it is possible that these values are more salient and more directly relevant to mental health behaviors than broader cultural goals in college students (extended discussion of the factor analysis and cultural values scales alone is available in the supplementary text).

### Shared Cultural Values, Perceived Need, and Help-Seeking

In the current study, we observed that students who scored highly on our IO factor were less likely to endorse needing mental health treatment, over and above ethnicity and their mental health attitudes. In interpreting this finding, it is important to consider research showing that familism and group-orientation, two subcomponents of IO, can act as protective factors for mental health [14]. In particular, group-oriented attitudes, such as a greater sense of pride and belonging to one’s ethnic group, have been linked to a decreased likelihood of psychiatric service use in Latinx adults and decreased odds of lifetime psychiatric diagnoses for both Asian and Latinx adults [62, 63]. It is possible that students in our sample with higher IO experience the benefits of familism and in-group orientation in having a greater support network and may thus be in lesser need of mental health services. However, in light of our finding that greater IO also predicted negative mental health attitudes, an alternative interpretation is that IO might encourage underreported or under-perceived need for help, on which critically important help-seeking behavior is contingent. This association was not observed with our CO factor. An important direction for future research will be to explicitly arbitrate between these possibilities. Our initial data from a fairly large and diverse college sample indicate that probing underlying shared cultural values, irrespective of ethnicity, may be informative for university mental health service providers. We therefore contend that probing cultural values when assessing students’ mental health needs is more in line with students’ lived experiences than relying on ethnic background alone.

Notably, IO inversely predicted perceived need, whereas the LVS alone did not, even though the IO factor was largely comprised of LVS items. We note that neither the retained AVS-R or LVS items separately were predictive of any outcomes of interest. As such, the IO factor contributed beyond the role of AVS and LVS examined separately and may thus be more relevant for predicting mental health behaviors in our sample. Our data indicate that probing underlying values that have been typically associated with specific ethnic groups outside of the bounds of ethnicity and culture may be informative for university mental health service providers.

### Limitations and Future Directions

Our findings should be interpreted with several limitations in mind. First, the ethnic demographic makeup of our university (33.8% Asian, 41.5% Latinx) is not representative of the larger US population (5.9% Asian, 18.5% Latinx) [64] and thus our findings may not be reflective of broader Asian and Latinx experiences—that is, those participating in our study are surrounded by more diverse cultural feedback than the average Asian or Latinx youth might be. Moreover, it is important to note that both ethnic groups are heterogeneous, and thus students may have varied more in their experiences and adherence to broad Asian or Latinx values specifically, which adds to our efforts to look broadly at shared themes rather than values specific to any one group. Second, college environments have uniquely accessible on-campus counseling centers with flexible service delivery and payment options, staffed by mental health professionals with specialized training to work with college youth and motivated to reach students. Thus, the mental health environment of college campuses does not accurately capture off-campus mental health experiences or other types of mental health help that Asian and Latinx youth receive. Third, given that only those students who indicated mental health treatment need reported on help-seeking behaviors, we were underpowered in predicting help-seeking behaviors—only 33 participants responded to this question. It is possible that students were reluctant to self-report on needing and seeking treatment, even though their answers were anonymized. Including implicit measures of emotional health to complement self-report may further our understanding of the broader sociocultural contexts in which youth develop [65]. Lastly, this was a cross-sectional study; a longitudinal design examining the interplay between interdependent cultural value endorsement, mental health attitudes, and help-seeking behavior over time would provide insights into the direction of effects of these variables.

Given the nuanced role that interdependent cultural values play in emotional well-being, it will be important to examine circumstances under which these group-oriented values may be beneficial or detrimental to mental health attitudes and behaviors. Under a cultural value-based approach, values that may otherwise seem irrelevant may actually hold significance—that is, it is important to strike a balance between understanding how individuals uniquely perceive their cultures and also considering intersectionality and inter-group similarities. For instance, research might probe whether greater adherence to *Interdependent Orientation* predicts negative mental health attitudes and mental health behaviors in other ethnic groups as well. Additionally, though the current work identified similarities in internal barriers to help-seeking behaviors across groups, we are in no way stating that structural barriers, such as diminished access to resources, or cost, are irrelevant to mental health behaviors in ethnic minority youth. We would also like to caution against an automatic preference for considering professional psychological services as the ideal method of support for mental health. Nonetheless, behavioral health services are the main source of mental health support offered by colleges and universities. As such, it is important to better understand why some students might not be engaging with these available services. If specific cultural values are leading students to underutilize campus mental health resources but perhaps engage in other types of help-seeking, then it is important to identify what alternative methods of support might look like. Doing so would also inform outreach programs to bring awareness to alternative resources available for student mental health. Future studies might build upon our findings and examine interactions between cultural values and resource availability. Next steps might focus on further parsing out the multifaceted experience of familism, taking a strength-based perspective in understanding when strong adherence to interdependent values may act as a buffer for mental health problems and the stresses unique to the university environment. By doing so, researchers may utilize and amplify the positive effects of forces already in play rather than “culture-blaming.”

## Conclusions

Asian and Latinx college students exhibit similarly low rates of mental health help-seeking behaviors, yet little work has examined what value-based similarities both groups might share that may contribute to these behavioral outcomes. The current study sought to test whether shared cultural values influence mental health help-seeking behaviors in ethnic minority students outside ethnic group membership alone. We focused on cultural values typically associated with specific ethnicities and considered the possibility of shared cultural values of diverse students together rather than separately. Our findings showed that Asian and Latinx students share values surrounding family, group, and culture. Further, their interdependent values influenced their attitudes towards mental health and influenced their perception of their own mental health treatment needs, a key step in seeking mental health help. Our findings shed light on the attitudes and behaviors regarding mental health help-seeking among both Asian and Latinx students and may inform future interventions and services in university contexts.

## Supplementary Information

ESM 1 (DOCX 23 kb)
