# Supplementary material for: Shared Cultural Values Influence Mental Health Help-Seeking Behaviors in Asian and Latinx College Students
Source: J Racial Ethn Health Disparities. 2021 Jun 23;9(4):1325–34. doi: 10.1007/s40615-021-01073-w (PMC9249685; doi:10.1007/s40615-021-01073-w)
Supplement: Supplementary file 1 — (DOCX 23 kb) [file 40615_2021_1073_MOESM1_ESM.docx]

**Supplementary Text**

**Factor Analysis**

***Methods***

Several criteria in determining factorability were met before conducting the factor analysis. The original scales, the AVS-R and LVS, were shown to be strongly correlated. The Kaiser-Meyer-Olkin measure of sampling adequacy was 0.74, and Bartlett’s test of sphericity was significant, χ^2^(1770) = 4072.18, *p* < .001. Finally, our factor analysis results showed that the communalities of 13 of the selected 14 items exceeded .3 [1]. In sum, the items together were an appropriate candidate for factor analysis.

Principal axis factoring was chosen as an extraction method to measure the latent commonality across the AVS-R and LVS given similar participant adherence to both scales [2]. A direct oblimin rotation was selected based on the correlations between scales to allow for possible correlation between factors [3]. Sixty-three iterations were necessary.

With the scree plot as an initial guide to determining the factors, it was noted that the bend of the elbow occurred after the first two factors. As such, only the first two factors were retained for the combined model. Of the items that loaded onto these factors, only reaching a threshold of factor loading .4 were deemed acceptable [4]. For those with cross-loadings, those items with loadings of .4 or greater on more than one factor were then either retained on the factor on which it more strongly loaded if the difference between the loadings met or surpassed .2, or otherwise dropped [4]. The two factors explained 24.6% of the variance, with *Interdependent Orientation* explaining 18.6% and *Cultural Obligation* explaining 6.07%.

***Results***

Our factor analysis of two standardized scales – the Asian Values Scale Revised [5] and the Latino Values Scale [6] – revealed two common factors underlying shared cultural values across our diverse college sample: *Interdependent Orientation* and *Cultural Obligation*. These two factors are comparable to factors that originally emerged in the creation of the LVS, *Familism* and *Cultural Pride*. However, the IO and CO factors which were observed in the current study contain different items, including two from the AVS-R: *one should not deviate from familial and social norms* and *one’s achievements should be viewed as family’s achievements*. Of note, even though both factors were derived from AVS-R and LVS, they were primarily driven by LVS items centering on family, group, and culture themes that have emerged in research on both Asian and Latinx individuals [7, 8]. The authors of the original AVS also found similar patterns to the ones that emerged in the current study and in the creation of the LVS, including values centered on: *Conformity to Norms, Collectivism,* and *Filial Piety* [9]. Importantly, this commonality across both the LVS and the AVS-R suggests that in the context of college students, values centering on interdependence and group-orientation may be a key to understanding commonality across mental health behavior regardless of cultural or ethnic group.

**Linear Regressions: AVS-R and LVS Predicting Attitudes towards Mental Health**

***Results***

First, using attitudes to mental health as the dependent variable in a stepwise regression, we entered ethnicity and gender, followed by the AVS-R, and lastly AVS-R × demographic variables (ethnicity, gender). Ethnicity and gender were significantly associated with attitudes towards mental health, *R*^2^ = 0.12, *F*(2,156) = 10.91, *p* < 0.001. AVS-R significantly added to the prediction of attitudes towards mental health, Δ*R*^2^ = 0.16, *F*(1,155) = 34.14, *p* < 0.001. Results showed that AVS-R was associated with dampened AMI total scores, after controlling for demographic variables. The step containing the interaction term with ethnicity and gender did not add significantly to the prediction of attitudes towards mental health (see Table S2).

Using attitudes to mental health as the dependent variable in a regression as with the AVS-R above, we entered ethnicity and gender on an initial step, the LVS on a second step, and LVS × demographic interaction terms on a third step. As above, ethnicity and gender were significantly associated with attitudes towards mental health, *R*^2^ = 0.12, *F*(2,156) = 10.91, *p* < 0.001. The step containing the LVS significantly added to the prediction of attitudes towards mental health, Δ*R*^2^ = 0.07, *F*(1,155) = 13.67, *p* < 0.001. The results indicated that LVS-R scores were a significant inverse predictor of the AMI total scores, after controlling for demographic variables. The step containing the interaction term with ethnicity and gender did not add significantly to the prediction of attitudes towards mental health (see Table S3).

***Discussion***

Adherence to Asian and Latinx cultural values inversely predicted positive attitudes towards mental health across all students irrespective of their ethnic backgrounds. That is, students who scored high on the AVS-R and the LVS were less likely to endorse positive attitudes towards people with mental health problems. These findings extend prior work in Asian and Latinx groups separately on stigma and negative attitudes to mental illness [10, 11, 12, 5]. Indeed, individuals from both ethnic groups indicate a greater motivation to avoid engagement with someone with a mental illness, though this has largely emerged from studying attitudes within each group individually [13, 14]. Considering the predictive power of these values on influential mental health attitudes may help bolster our understanding of shared mental health outcomes.

**Supplementary References**

1. Neill, J. (2008). *Writing up a factor analysis*. Retrieved from http://www.bwgriffin.com/gsu/courses/edur9131/content/Neill2008_WritingUpAFactorAnalysis.pdf
2. De Winter, J., Dodou, D., & Wieringa, P.A. (2009). Exploratory factor analysis with small sample sizes. *Multivariate Behavioral Research*, *44*(2), 147-181. https://doi.org/10.1080/00273170902794206
3. Costello, A.B. & Osborne, J.W. (2005). Best practices in exploratory factor analysis: Four recommendations for getting the most from your analysis. *Practical Assessment, 10*(7), 1-9. https://doi.org/10.7275/jyj1-4868
4. Matsunaga, M. (2010). How to factor-analyze your data right: Do’s don’ts, and how-to’s. *International Journal of Psychological Research, 3*(1), 97-110. <https://doi.org/10.21500/20112084.854>
5. Kim B. S., Omizo M. M. (2003). Asian cultural values, attitudes toward seeking professional psychological help, and willingness to see a counselor. *The Counseling Psychologist*, *31*(3), 343–361. <http://dx.doi.org/10.1177/0011000003031003008>
6. Kim, B. S. K., Soliz, A., Orellana, B., & Alamilla, S. G. (2009). Latino/a Values Scale: Development, reliability, and validity. *Measurement and Evaluation in Counseling and Development*, *42*(2), 71–91. <https://doi.org/10.1177/0748175609336861>
7. Leong, F. T. & Lau, A. S. (2001). Barriers to providing effective mental health services to Asian Americans. *Mental Health Services Research*, *3*(4), 201–214. <https://doi.org/10.1023/a:1013177014788>
8. Ramos-Sánchez, L., & Atkinson, D. R. (2009). The relationships between Mexican American acculturation, cultural values, gender, and help-seeking intentions. *Journal of Counseling & Development*, *87*(1), 62–71. <https://doi.org/10.1002/j.1556-6678.2009.tb00550.x>
9. Kim, B. S. K., Atkinson, D. R., & Yang, P. H. (1999). The Asian Values Scale: Development, factor analysis, validation, and reliability. Journal of Counseling Psychology, 46(3), 342–352. <https://doi.org/10.1037/0022-0167.46.3.342>
10. Abdullah, T., & Brown, T. L. (2011). Mental illness stigma and ethnocultural beliefs, values, and norms: An integrative review. *Clinical Psychology Review*, *31*(6), 934–948. <https://doi.org/10.1016/j.cpr.2011.05.003>
11. Han, M., & Pong, H. (2015). Mental health help-seeking behaviors among Asian American community college students: The effect of stigma, cultural barriers, and acculturation. *Journal of College Student Development*, *56*(1), 1–14. <http://dx.doi.org/10.1353/csd.2015.0001>
12. Interian, A., Martinez, I. E., Guarnaccia, P. J., Vega, W. A., & Escobar, J. I. (2007). A qualitative analysis of the perception of stigma among Latinos receiving antidepressants. *Psychiatric Services*, *58*(12), 1591–1594. <https://doi.org/10.1176/appi.ps.58.12.1591>
13. Whaley, A. L. (1997). Ethnic and racial differences in perceptions of dangerousness of persons with mental illness. *Psychiatric Services*, *48*(10), 1328–1330. <https://doi.org/10.1176/ps.48.10.1328>
14. Yang, L. H., Purdie-Vaughns, V., Kotabe, H., Link, B. G., Saw, A., Wong, G., & Phelan, J. C. (2013). Culture, threat, and mental illness stigma: Identifying culture-specific threat among Chinese-American groups. *Social Science & Medicine*, *88*, 56–67. <https://doi.org/10.1016/j.socscimed.2013.03.036>
